# Challenges in primary care for diabetes and hypertension: an observational study of the Kolar district in rural India

**DOI:** 10.1186/s12913-019-3876-9

**Published:** 2019-01-18

**Authors:** Dorothy Lall, Nora Engel, Narayanan Devadasan, Klasien Horstman, Bart Criel

**Affiliations:** 1 Institute of Public Health, 2nd Cross, Girinagar, 1st Phase, Bengaluru, 560085 India; 2 Department of Health, Ethics & Society, CAPHRI Care and Public Health Research Institute, P.O. Box 616 6200, MD Maastricht, The Netherlands; 3 Institute of Tropical Medicine, Nationalestraat 155, 2000 Antwerpen, Belgium

**Keywords:** Primary care, Diabetes, Hypertension, LMIC, Organisation, Care, Health services, Chronic care, Challenges

## Abstract

**Background:**

Chronic diseases have emerged as the leading cause of death globally, and 20% of Indians are estimated to suffer from a chronic condition. Care for chronic diseases poses a major public health challenge, especially when health care delivery has been geared traditionally towards acute care. In this study, we aimed to better understand how primary care for diabetes and hypertension is currently organised in first-line health facilities in rural India, and propose evidence-based ways forward for strengthening local health systems to address chronic problems.

**Methods:**

We used qualitative and quantitative methods to gain insight into how care is organised and how patients and providers manage within this delivery system. We conducted in-depth interviews with the medical doctors working in three private clinics and in three public primary health centres. We also interviewed 24 patients with chronic diseases receiving care in the two sub-sectors. Non-participant observations and facility assessments were performed to triangulate the findings from the interviews.

**Results:**

The current delivery system has many problems impeding the delivery of quality care for chronic conditions. In both the public and private facilities studied, the care processes are very doctor-centred, with little room for other health centre staff. Doctors face very high workloads, especially in the public sector, jeopardising proper communication with patients and adequate counselling. In addition, the health information system is fragmented and provides little or no support for patient follow-up and self-management. The patient is largely left on their own in trying to make sense of their condition and in finding their way in a complex and scattered health care landscape.

**Conclusions:**

The design and organisation of care for persons with chronic diseases in India needs to be rethought. More space and responsibility should be given to the primary care level, and relatively less to the more specialised hospital level. Furthermore, doctors should consider delegating some of their tasks to other staff in the first-line health facility to significantly reduce their workload and increase time available for communication. The health information system needs to be adapted to better ensure continuity of care and support self-management by patients.

**Electronic supplementary material:**

The online version of this article (10.1186/s12913-019-3876-9) contains supplementary material, which is available to authorized users.

## Background

Chronic diseases and conditions, such as heart disease, diabetes, stroke, and cancer, have emerged as the most common and costly of all health problems worldwide [1]. Similar to other low- and middle-income countries experiencing an epidemiological transition [2], India has witnessed an escalation of chronic diseases in recent years [3]. An estimated 20% of Indians suffer from a chronic condition [4], and in 2017 there were 72 million persons living with diabetes in India [5], and an estimated 200 million with hypertension [6].

Care for chronic diseases poses a real challenge for the health care delivery system, especially in low- and middle-income countries like India [7–9], as the requirements of chronic care differ from acute episodic care, which health systems are traditionally geared towards [10]. Chronic diseases have various definitions, conveying that these are conditions of long duration, often with a long latency period and protracted clinical course, of multi-factorial aetiology with no definite cure, and characterised by gradual changes over time [11]. These characteristics necessitate sustained engagement of the health system with over the lifespan of the individual, continuity and coordination of care across health care levels and health specialities, and recognition of the person at the centre of the care process [10]. Primary health care has been envisaged as the level of care at which all of the demands of chronic care can be meaningfully met [12] to prevent and control disease in an equitable manner [13].

The Chronic Care Model (CCM) was developed in the 1990s from a robust review of the literature as a framework for the organisation of services for chronic conditions at the primary care level [14]. This model is based on the premise that good clinical outcomes result from productive and meaningful interactions between patients and their health care team. To achieve productive interactions, the health care system needs to have four developed areas at the level of the health facility: self-management support, delivery system design, decision support, and clinical information systems along with links in the community. The concept of self-management [15] calls for greater involvement and participation of patients and their families in caring for the condition. A person-centred approach with a focus on communication and relationships was also identified in the Institute of Medicine’s landmark report on the quality chasm in health care as being essential to good quality of care [16].

In India, health care at the primary level is the backbone of health service delivery, which is structured across a three-tiered system (primary, secondary, and tertiary services). India has a mixed health care system with public and private sectors that are governed at the state level, based on a federal structure, and the centre [17] through programs and policies. The private sector has increased rapidly over the last few decades; currently, more than 70% of health care is in the private sector [18]. Poor regulation of the private sector by the government and poor delivery of services through government structures have contributed to a weak and fragmented health system [19, 20]. In this context, care for diabetes and hypertension remains suboptimal [21] with many systemic barriers, such as poor access to medicine and lack of workforce to deliver services at the primary care level [22].

A recent assessment of resource availability at primary care centres in rural India, concluded that there was a lack of preparedness to deliver comprehensive care for diabetes at this level of health care delivery [23]. However, there is a gap in the literature regarding how patients and providers cope with these systemic issues in daily practice and how they specifically impact the care of chronic conditions, especially in the private sector.

Here, we sought to understand the organisation of care for diabetes and hypertension at the primary health care level in both the private and public sectors in a rural district of India. We use the CCM to make sense of provider and patient experiences, perspectives, and expectations as they cope with diabetes or hypertension in the current delivery system.

## Methods

We aimed to to identify and recommend solutions for better care of persons with a chronic condition by determining how care for diabetes and hypertension is organised in public and private sector structures of primary health care in a rural district, as well as the challenges faced by patients and providers and how they cope within the current organisation of the delivery system.

### Study design

We used both qualitative and quantitative methods to understand the process of care from both the patient’s and provider’s perspective. The qualitative methods comprised in-depth interviews and non-participant observations to understand practices and ways of coping within this delivery system. The quantitative methods comprised a health facility assessment (checklist) to triangulate the findings obtained with the qualitative methods.

### Study setting and context

The study was conducted in Kolar, a rural district in the southern state of Karnataka, India. The district has a total population of 1,536,401 according to the 2011 census [24]. In this district, health care services for diabetes and hypertension are delivered in both private and public health sectors. In the public sector, the services are governed by the Department of Health for Karnataka state [25] and delivered at District Hospital, Community Health Centres (CHCs), Primary Health Centre (PHCs), and Sub-Centres (SCs). The SCs and PHCs are the primary level of the health care infrastructure. The PHC is the first level of care with a medical doctor and each usually covers a population of 30,000. Kolar district has 60 PHCs across its five taluks (sub-district division). There is one community health worker (ASHA) for every 1000 people, who is largely involved in maternal and child health service delivery. The CHCs, one for every 80,000 people, is the first referral unit equipped with 30 beds and an operating theatre with multiple speciality services. The district hospitals are larger with more specialities and facilities and range from 100 to 500 beds. In Kolar, there are more than 250 private health facilities of varying infrastructure and specialities, including practitioners of the Indian Systems of Medicine, outnumbering the public health facilities by many fold.

The government of India launched a specific programme for the control of diabetes, cancer, and stroke in 2009, the NPCDCS (national program for control of diabetes, cancers and stroke). The programme is largely vertically structured, managed by state governments, but guided by national programme policies. The programme has created NCD (non- communicable disease) cells at CHCs and district hospitals to provide clinical care for diabetes and hypertension [26]. The role of NCD care at the PHC level (see Table 1) is minimal. According to the programme, persons diagnosed with diabetes or hypertension should be referred to the NCD cell for confirmation, probably due to the lack of laboratory facility, as venous blood glucose estimation at the PHC is required to diagnose diabetes. The NPCDCS has been implemented in 100 of the 700 districts all over the country during the pilot phase and is now being expanded to other districts. Kolar is one of the pilot districts of the programme, in operation since 2009.

**Table 1:** Package of services available under NPCDCS at various levels of health care [26]

Level of health care in the system	Staff that should be available at this level of health care according to guidelines	Package of services under NPCDCS
Sub-Centre	One auxiliary nurse midwife (ANM) One male health worker [40]	1) Health promotion 2) ‘Opportunistic’ screening for diabetes and hypertension 3) Referral of suspected cases to CHC
Primary Health Centre (PHC)	One medical officer, three nurses, midwife, health worker, pharmacist, lab technician [25]	1) Health promotion 2) ‘Opportunistic’ screening for diabetes and hypertension 3) Clinical diagnosis and treatment of simple cases of hypertension and diabetes 4) Referral of cases with suspected diabetes or hypertension for confirmation to CHC
Community Health Centre (CHC)	Medical superintendent, five speciality doctors, one duty officer, 10 staff nurses, one pharmacist One medical officer, one nurse, one counsellor [41]	1) Prevention and health promotion 2) Early diagnosis 3) Management of common CVDs, diabetes, and stroke 4) Referral of difficult cases to District Hospital
District Hospital	18 speciality doctors, 11 medical officers, 45 staff nurses, 6 lab technicians, 4 pharmacists One general physician, two nurses, one technician, one physiotherapist, one counsellor, and one data entry operator [42]	1) Early diagnosis of diabetes, CVDs, and cancer 2) Medical management of cases (outpatient, inpatient, and intensive care) 3) Referral of difficult cases to higher health care facility 4) Health promotion 5) Rehabilitation and physiotherapy services

*CVDs* cardiovascular diseases

### Study participants and sampling

We conducted in-depth interviews of patients with diabetes, hypertension, or both. These patients were stratified according to their health-seeking behaviour as those seeking care from PHCs or a private health facility, and those not seeking care from a public or private health facility in the last 6 months, defined as “not in regular care” for the purpose of this study.

Those seeking care from PHCs or a private health facility were contacted at the health facility and sampled consecutively on the day the investigator was at the health facility. The patients willing to participate in the study were introduced to the investigator by the treating physician. Four patients at these health facilities (3 at public facilities and 1 at the private facility) did not want to be interviewed, citing a lack of time.

The patients not in regular care were identified from a survey of patients diagnosed with diabetes or hypertension. These patients had been identified by the NPCDCS during routine programme activities. This list of patients was obtained from the government district health office and 100 were systematically stratified, taluk wise, and selected for the survey. Each person was asked where they had sought care for diabetes or hypertension in the last 6 months. Ten (10%) reported that they had not sought care at any health facility in the last 6 months (not in care), 44% reported seeking care at a private facility, and 46% at a public facility. We interviewed respondents in each category, 24 attending a private health facility, 12 attending a public health facility, and 6 not in care. In each category, we recruited participants consecutively until the responses were very similar (i.e., data saturation was achieved).

We also conducted in-depth interviews with doctors at three PHCs and three private health facilities. The PHCs were randomly selected from a list of eligible PHCs in Kolar, defined as PHCs in the district that delivered services for people with diabetes and hypertension, with a medical doctor and basic infrastructure. We could not obtain a list of private health facilities that indicated whether the facility provided care for diabetes and hypertension. Therefore, we sampled the facilities based on the responses to the survey, choosing the three most popularly reported private health facilities.

### Data collection

We conducted semi-structured in-depth interviews with 24 patients and 6 providers at the sampled health facilities from Oct 2016 to Feb 2017. All patients selected from the health facility preferred to be interviewed in a private location at the health facility. In 10 of the 24 interviews, a family member accompanying the patient was present during the interview. Persons not in care were interviewed at their homes, and four of the six interviews had a family member present. The interview guides were developed using the CCM as a framework and pre-tested (Additional file 1). The guides were refined throughout the data collection period in an iterative manner based on interpretation and analysis as data were collected. The interviews were conducted in either the local language (Kannada and Hindi) or English based on the convenience of the respondent by the first author and a public health researcher, both trained in qualitative research methods. Each interview lasted 30 min on average. The interviews conducted in Kannada were translated into English and transcribed verbatim by a professional. Interviews conducted in Hindi and English were also transcribed verbatim by the first author.

In addition, the primary author conducted non-participant observations focusing on processes of care at each facility in the consulting rooms, waiting areas, pharmacy, and laboratory during the outpatient consulting hours (6 h in private facilities and 4 h in public facilities) using a semi-structured guide (Additional file 2). The observations involved detailed note-taking of structures, persons, and interactions between health staff and patients that lasted for an average of 1 h in each area. We also conducted assessments for each health facility and assessed health personnel, supplies, and equipment using a pre-tested structured data collection tool (Additional file 3) based on the Indian guidelines for PHCs and the World Health Organisation essential package of services for NCDs [25, 27]. The observations and health facility assessments were conducted before the interviews, providing us with the opportunity to clarify or probe specific observations. The quantitative data were used to triangulate the data collected in the interviews.

### Data analysis

The transcripts of the interviews were analysed thematically in Nvivo qualitative data analysis software (QSR International Pty Ltd., version 11, 2015). The participants’ personal details were removed and audio files anonymised to maintain confidentiality. The data were initially coded into pre-specified codes based on the elements of the CCM: delivery design, information systems, decision support, self-management support, community links, and organisation of care. New codes were created to describe the data that did not fit these pre-specified codes, such as communication during the care process and resource availability.

Coding was done by the primary author and memos were shared and discussed with the team to refine the codes. Dominant themes in the coded data were identified through analysis of text, repetitive reading and comparison with literature. These were tested, and refined through an iterative process with other members of the team (Additional file 4). Patient and provider interviews were included in the same dataset for coding. The emerging themes were then tested across the different sources of interviews (patient, doctors and other health staff) for consistency.

The facility assessments and non-participant observations were used to triangulate the findings from the interviews to enhance internal validity. Descriptions of the non-participant observations were uploaded to a Nvivo database and were thematically analysed to look for inconsistencies or contradictions with the themes of the interviews. Facility checklists were used for verification of the themes, such as, the theme of availability of resources could be directly verified by availability of resources recorded in the checklist at the time of the interviews. There were no inconsistencies found in the three sources of data.

The major themes identified were doctor-centered care processes that impacted communication, fragmented care processes impacted by a reality of scarce resources, decision making for care that was not evidence based nor patient-centred, broken information systems that was a challenge to continuity of care and the work done by patients in “self-management” and managing care processes.

The doctors at the three private health facilities had been practising in the area for more than 30 years and were > 50 years of age. They also had medical degrees up to Masters in General Medicine. Two of the three private providers received additional training in diabetes management and had certification displayed in their consulting rooms. The doctors at the PHCs were younger (< 35 years of age) and had been practising for 3 to 7 years. They were trained in modern medicine up to a graduate level (Table 2).

**Table 2:** Characteristics of doctors in charge at health facilities

No.	Type of health facility	Sex of doctor	Age (years) of doctor	Medical qualifications	Years in practice
1	Private Clinic 1	Male	50–60	Medical doctor with post-graduate in general medicine	20–30
2	Private Clinic 2	Male	50–60	Medical doctor with post-graduate in general medicine	20–30
3	Private Clinic 3	Male	60–70	Medical doctor with post-graduate in general medicine	20–30
4	PHC 1	Female	20–30	Medical doctor	1–10
5	PHC 2	Female	20–30	Medical doctor	1–10
6	PHC 3	Male	20–30	Medical doctor	1–10

The three private health facilities were out-patient clinics, and one had an in-patient facility with 15 beds (Private clinic 1). All three facilities had pharmacies within the same location, and two of the three facilities also had laboratory services. The three PHCs were similar with respect to infrastructure and the services they provided. All of the facilities had basic equipment and essential medications (Additional file 5) at the time of assessment. The majority of the 18 patients interviewed at the health facilities were female (61%) and most of them had diabetes (Table 3).

**Table 3:** Characteristics of patients recruited for in-depth interviews

Type of facility	Age range (years)	Gender	Time elapsed since diagnosis	Condition
Private (*n* = 6)	42–69	43% F	6 months - 15 years	71% DM, 29% HTN + DM
Public (*n* = 12)	36–73	88% F	9 months – 25 years	55% HTN, 45% DM
Not in care (*n* = 6)	42–60	66% F	2–6 years	83% DM, 17% HTN

*DM* diabetes, *HTN* hypertension, *HTN + DM* hypertension and diabetes

## Results

We present the findings within each theme from both the patients’ and providers’ perspectives to enable a comprehensive understanding of the processes involved in seeking and delivering care. We present the findings for the three private clinics first, and then for the three public health facilities.

### Doctor-centred care process impacting communication

At both public and private health facilities, patients start their journey through the health facility by meeting the doctor. The number of consultations were limited, by use of tokens, at private health facilities, usually up to 50 during 6 h of OPD compared to an average of 75 patients (up to 150) in 4 h at public health facilities. All of the interactions that follow at the laboratory or pharmacy were centred around and informed by the interaction with the doctor.

*“We will first go to the doctor, he will give the prescription for the tests, then we will come to the nurse and get the blood test done for sugar levels, and get the BP check -up done, if they say it is normal, we will go back to doctor and take the tablets which are in stock and come back”. (Patient 1 at private health facility)*

This may be due, at least in part, to the expectation patients have of being seen and advised primarily by doctors, but also because of the prominent hierarchy that exists between doctors and other health professionals, with the former at the apex. There was a lack of specified and planned interactions with other members of the team, such as the nurse or lab technician.

*“sometimes staff nurse will be there counselling and she may be involved, suppose…. they are having severe hypertension or hypertension at present or diabetes at present I will send them to the sister just to counsel them, if I am free I will be talking to the patient if I am not free or a little busy I will ask the sister and I will say please explain this patient what about his diet and role of the diet or exercise and all”. (Provider 6 at Private facility)*

This placed the burden of examining, screening for risk factors or complications, treating, sharing information of lifestyle modification, and ensuring follow-up entirely on the doctor. Within the few minutes the doctor spends with the patients, it was not always possible to complete many of these tasks, such as screening for risk factors, complications, and support for lifestyle modifications. Patients especially in the public health facilities were dissatisfied with the time the doctor spent.

*“they are not interested to hear they don’t listen, the doctor doesn’t have time...if we have much to say they say come to my hospital (doctors private practice)” (Patient 5 at public facility)*

Notably, at both public and private facilities, the process of care was not any different for persons with chronic conditions, such as diabetes or hypertension, than persons with acute conditions.

### Fragmented care processes impacted by a reality of scarce resources

In all three private facilities, a pharmacy was available at the same location, however, a laboratory was present in only 2 of the 3 facilities. Mostly, patients were able to have their blood tested and procure medicines in the same facility but when then this was not the case, patients found this quite challenging. Especially, as it involved finding a suitable laboratory and an additional visit to the laboratory or pharmacy.

“*There is no lab here, that is the problem, we have to go 7 km away, we have to go in the morning and give the sample and again after breakfast we have to go after 2 hours, and by the time we get the report, it will be half a day, and again the next day to show that to doctor we have to spend half day, so by this we will be wasting our 2 days.” (Patient 16 at private health facility)*

In the public facilities, laboratory and pharmacy were available at the same location, but these services were often unavailable due to a lack of supplies and medicines.

“*I will go to [the town of] Mulbagal to get the medicines as they are not available and it is 10 km away from my place.” (Patient 11 at public health facility)*

*“Not only in our PHC, but like other PHCs, also District Hospital and Taluka Hospital, [the medicine] was not there…for more than 6 months.” (Provider 5 at Public facility)*

*“the fasting and the one after food (blood tests) are not being done here so they go for SNR govt hospital (District hospital) and there it will be done….they do there and they will get opinion from the physician…..what to be done what not to be done and we continue it here”- (Provider 3 at public facility)*

PHC doctors perceived their role reduced to merely pursuing the treatment process started by the physician at the NCD cell. This is in line with the limited role and package of services attributed by the NPCDCC to the primary care level.

### Decision-making for care- not evidence- based nor patient- centred

At the private health facilities, doctors made treatment decisions based only on blood glucose levels. Another, important consideration was the patient’s economic ability to afford medication. The decision to screen for complications and perform further investigations was also determined by their ability pay for it.

*“I do a rough socio-economic assessment and prescribe what I think they can afford.” (Provider 6 at a Private facility)*

*“So, in the initial assessment we do all the parameters. If they can’t afford [more], only blood sugar will be done, but if they can afford [it], all the parameters will be done.” (Provider 2 at Private facility)*

Doctors feel that patients should not be involved in decision-making on their treatment and feel that they do not have the knowledge to meaningfully participate.

*“This must never be done [asking patients about preferences], you have to tell the patient and not allow them to tell you what to give or not. Not in a bad way that I am the doctor and you are the patient, not that attitude, but explaining nicely.” (Provider 6 at Private facility)*

*“awareness is a big challenge, “they (patients) don’t understand and in diabetes they become forgetful also and don’t remember the advice”- (Provider 1 at Private facility)”*

Also, at the PHCs, doctors did not involve patients in treatment decision-making, perhaps because of a lack of time. Most patients did not expect them to do so.

*“Whatever he writes I just take and come back.” (Patient 6 at public facility; referring to the prescription that the doctor gives at the end of consultation)*

Doctors at private facilities found treatment guidelines and protocols difficult to apply. Two of them said that, though they were aware of international guidelines, such as those issued by the American Diabetes Association (ADA), they did not follow them completely and thought they were not practical. This may be due to a lack of information regarding risk factor status or complications, which is required in order to follow these treatment algorithms but not recorded at any of the facilities. However, the doctors seem to be guided by other social and contextual factors that, in their experience, influence compliance and regular follow-up.

*“I start with metformin like ADA says to start with, but if not controlled I don’t increase as much as ADA says, but add a gliptin [class of antidiabetic drug], and nowadays I prefer the 4GPPs [newer class of antidiabetic drugs].” (Provider 6 at Private facility)*

At the public health facilities, doctors also made treatment decisions based on the blood glucose levels, usually random blood glucose using a point of care device. The doctor stocks medications that are on the essential drug list and has a choice of usually only two (metformin and glibenclamide). None of the three doctors at the PHCs were aware of any treatment guidelines provided by the NPCDCS [28] that provide guidance on medication choice and dosage.

“No written guidelines or algorithms are provided to us.” (Provider 4 at Public facility)

Other than blood glucose levels, treatment decisions are guided by affordability in private facilities and mainly by the availability of medicine in the public facilities. However, neither public nor private facilities follow guidelines; they lack information to help guide decisions, even if there is some awareness of guidelines and attempts to use them by the private doctors.

### Broken health information systems: A challenge to continuity of care

The private health facilities encourage the patient to retain a notebook containing the medication and last blood pressure or glucose recordings. However, no recording of patient details is maintained at any of the health facilities, placing the responsibility to bring this information for continuity of care completely on the patient.

“*I usually ask them to bring a notebook with them and record in this each time.*” (*Provider 1 at private faciity)*

The doctors do not have information available to identify high risk patients, schedule follow-up visits, and ensure regular care, resulting in patients dropping out of care.

*“They told [me] the sugar levels are normal and to take the same tablets, so I did not go.” (Patient 17- not in regular care but previously seeking care at a private health facility)*

At public health facilities, the use of a notebook or a book given by the NPCDCS seems to be common practice. Even though such a patient-retained medical record is a laudable attempt at maintaining continuity, patients do not consistently use it. The patients are unable to appreciate its role in maintaining continuity, and many feel that the last prescription or empty blister pack was enough for this purpose because it was only important for the doctor to know what medication was prescribed.

*“The nurse and the doctor who gave me that book have changed so I don’t bring it anymore.” (Patient 14 at public health facility)*

Even though the PHCs have to submit monthly reports to the NCD cell, only total numbers screened and attendance at OPD are monitored. The lack of patient information results in all patients being asked to come back after 1 month, leading to more visits than may be necessary, which is both inconvenient for patients and increases patient load at the PHC.

The lack of recorded clinical information, the limited time for consultation, and the reluctance to involve patients in management decisions impacts the ability to use evidence-based guidelines and affects the quality of care provided.

### Work done by patients in “self- management” and managing care processes

Patients accessing care at public or private health facilities actively try to cope within this complex, fragmented health care delivery system. The CCM defines self-management as tasks that include taking medication, being aware of complications and danger signs, maintaining regular follow-up, and making sustained lifestyle changes. We found that patients have to do much more than this in the Indian context, including finding a health facility from which to seek care. Patients continuously try to make sense of their health condition, attempting to understand when their glucose levels or blood pressure is increased, why they got the condition, what food they need to eat, where to get tested, where to buy medication, and when to go back for a visit.

“*I will come to know, I will feel tired, and I will not get complete sleep.” (Patient 1 at a public health facility, regarding revisiting the health facility)*

*“If I feel the BP has gone up, if I have a headache, I will increase to two tablets a day, one in the morning and one in the night.” (Patient18 at public health facility)*

Most patients make lifestyle changes and are able to sustain them, especially in regarding their diet. The information they have comes from many sources other than the health facility, most commonly from others in the community.

*“Yes, less salt… I don’t put salt in the dough for roti [bread], salad also less salt, some say it is bland, but we have got used to it now.” (Patient 5 at private facility)*

“*We will be seeing people with diabetes in the society around us, and know to some extent how the diabetes patients should follow the diet.” (Patient 3 at private facility)*

We found that patients have to do much more than managing the disease condition, in the Indian context, including finding a health facility from which to seek care. Most of the patients had been to more than one provider, some up to five, since their condition was diagnosed. The health facilities they visited were a mix of public and private health facilities in Kolar and Bengaluru, the capital city of the state. When asked how they decide where to continue treatment, most expressed that the doctor speaking to them respectfully was an important consideration. Suggestions from family members and distance from home were other factors affecting choice of doctor.

*“They give good treatment and treat patients nicely, the treatment they give, we are getting good results” – (Patient 6 at private facility)*

In contrast, patients choose to attend a public health facility mainly because services, including medications, are available free of cost and they are close to where they live. However, if they had resources, they would prefer to seek care at private facilities because they perceive them to be better than public facilities.

Patients commonly expressed how common the disease was and how, if they continue to take their medicine, they would be “normal”. They seem to use sharing a condition with a large number of people as a mechanism to cope and make sense of living with a chronic condition.

*“I said to myself that this disease [diabetes] has become very common in India, we should take treatment properly to be fine.” (Patient 2 at private health facility)*

*“We have to bring down our disease, it is in our hands.” (Patient 7 at a private health facility*)

The fragmented delivery of services and the broken information system compel patients to do much more than just manage their disease, as it obviously has an impact on the entire process of care.

## Discussion

This study contributes to a better understanding of the processes of care for diabetes and hypertension in both public and private health facilities in a district of rural India. The results provide insights into how constraints within the health care delivery system impacts chronic care and how patients and providers cope with these constraints. We identified several challenges in the current organisation of services that impede the delivery of care responsive to the needs of chronic conditions, such as doctor-centred care processes that impede adequate counselling and result in poor communication, lack of decision support to enable evidence-based treatment decisions, a broken information system that does not facilitate follow-up and revisits, a fragmented delivery of services that requires multiple visits, and poor support for self-management that goes beyond just managing the disease condition.

Though the organisation of the care process was similar in both public and private health facilities, we found that both of these settings have their own unique challenges. While there is a flexibility in private clinics to limit the number of patients seen in one day and, thus, relatively more time to spend with patients compared to public clinics, it is still not adequate to consistently complete all of the tasks required for patient care. In both public and private health facilities, the current organisation of care processes is centred around the interaction with the doctor. This places the burden to complete most tasks in patient care, including providing information regarding lifestyle modification, on doctors. Task sharing is a solution that has been tested [29, 30] to overcome this constraint but is seldom seen in practise, perhaps due to hierarchies among health professionals, among other reasons [31]. This hierarchy is also reflected in the doctor-patient interaction, where we found that patients being involved in treatment decisions was not acceptable to doctors and not expected by patients. Patient-centred care, defined as “providing care that is respectful of and responsive to individual patient preferences, needs, and values and ensuring that patient values guide all clinical decisions” [32, 33], is poorly understood and practised in India.

Due to the prolonged nature of chronic conditions, it may be more important that patients and their families actively participate in the care of chronic conditions, referred to in the literature as self-care or self-management, and there are activities or tasks the patient should do to maintain their health [16]. These tasks include taking medication, being aware of complications and danger signs, maintaining regular follow-up, making sustained lifestyle changes, and managing emotional changes. However, we found that “self-management” in the Indian context goes far beyond this description. Patients have to find a suitable provider, plan their revisits, arrange transportation, and locate a laboratory and pharmacies that suit their location and finances in addition to the tasks mentioned above. The fragmented services, such as laboratory testing or pharmacy services, require multiple visits, travel across long distances, and considerable time at the health facility. Bhojani et al. explored constraints urban patients face in seeking care for NCDs at the primary care level in India and reported that this is a distressing challenge for most patients [34]. The difficulties navigating the Indian health system, especially for diagnosis, were also described by Yellapa et al., as patients often have to consult multiple providers across different health care levels, leading to frustration and delays in diagnosis [35].

Most models of chronic care, including the CCM, have support of self-management skills as an essential component of chronic care, but what this entails requires redefinition to suit the Indian context. As Starfield stated [12], when people and not diseases are the focus of our care, outcomes will be better and populations healthier. There is a paucity of literature from India investigating the work that patients have to do in self-management [36] and insufficient knowledge of how health providers can provide support.

Central to maintaining a sustained engagement are good information systems that record information about a patient’s health, identifying risk and using this information to inform treatment decisions and plan follow-up visits. In the absence of an information system, care for patients with a chronic condition ends up being multiple unconnected visits by a patient to the doctor, impacting care much more than in the case of an acute condition. In our study, we found that the information system was broken in both the public and private sectors, which was also reported by Bhojani et al. in their study on challenges of care in urban India and Mendis et al. in their study of the gaps in service delivery across several low- and middle-income countries [7, 22]. The use of patient-retained medical records seems a practical solution; however, without supplementing these records with information at the health facility, the entire responsibility of maintaining continuity of care is placed on the patients’ shoulders.

The lack of decision support to make evidence-based treatment decisions was a significant challenge for both the public and private sectors. In the public sector, there seems to be a greater need for strengthening capacity to follow the available guidelines, whereas in the private sector, communicating uniform guidelines could encourage their use to support decisions.

Another significant challenge in the delivery of services for diabetes and hypertension, particularly in the public sector, was a lack of resources in terms of a steady supply of medications, health personnel, and laboratory supplies. This is consistent with health facility assessments conducted in PHCs in Madhya Pradesh [23]. The NPCDCS envisions a very nominal role for chronic care at the primary health care level [26]. PHCs are expected to carry out opportunistic screening and manage simple cases of diabetes or hypertension, referring most patients to the NCD cell created by the programme at Taluk Hospital. However, PHCs are managing diabetes and hypertension and are capable of delivering care, as observed in a recent study that evaluated the integration of care for NCDs at PHCs and concluded that it was feasible to deliver NCD care at PHCs [37]. Globally, and specifically in other low- and middle-income countries, primary care has contributed to better health outcomes [38], and its role in the NPCDCS programme could be expanded.

Many of the challenges we identified can be attributed to a weak delivery of primary care with systemic impediments, such as poor information systems and lack of resources. Therefore, even though we studied only one district in rural India, the lessons we learnt may have implications for the organisation of service delivery, for diabetes and hypertension patients in the whole of rural India. The use of a chronic care model to reorganise services may be useful to address some of these systemic barriers and guide future action. It indeed, provides a reference framework that can be used to understand and treat health problems [39]. However, the CCM may need to be reconfigured to the realities of a health system in a middle-income country like India. We found that each of the CCM elements impacts and shapes the others, such as information systems impacting decision support and self-management support. This would need to be accounted for and these connections should be made more explicit in a model relevant to India.

## Conclusions

There is a need to better organise service delivery and care for patients with chronic conditions in rural India, especially within the framework of the national program. The service delivery system needs to encourage better communication to enable care centred around patients and their families, supporting them and responding to their needs. We recommend sharing tasks among health workers so that the care process is less dependent on the doctor, thereby freeing doctors’ time to address complex cases and to communicate with patients. Strengthening information systems will go a long way in enabling the scheduling of visits that do not overburden the doctor and are convenient for patients. It will also enhance evidence-based treatment decisions tailored to patient needs. The relevance and need to field test these changes in the organisation of health care delivery for chronic patients seems paramount.

## Additional files

Additional file 1: Interview guides. This document includes guides that were used to semi structure the in-depth interviews with doctors and patients. (DOCX 21 kb)

Additional file 2: Observation guide. This was a guide used to provide structure to the non-participant observations. (DOCX 15 kb)

Additional file 3: Facility assessment tool. This tool was created using documents (WHO Essential package of services and the Indian public health standards) that describe minimum available resources that should be available at primary level care. (DOCX 102 kb)

Additional file 4: Coding tree. This is the coding that was used in analysing the data. (DOCX 107 kb)

Additional file 5: Facility assessment. This provides the details of the assessment done for each health facility in the study. (DOCX 120 kb)

## Abbreviations

ASHA: Accredited Social Health Activist
CCM: Chronic Care Model
CHC: Community Health Centres
CVD: Cardiovascular Diseases
NPCDCS: National Program for Control of Diabetes, Cancers and Stroke
PHC: Primary Health Centre
SC: Sub-Centres
