# Supplementary material for: Challenges in primary care for diabetes and hypertension: an observational study of the Kolar district in rural India
Source: BMC Health Serv Res. 2019 Jan 18;19:44. doi: 10.1186/s12913-019-3876-9 (PMC6339380; doi:10.1186/s12913-019-3876-9)
Supplement: Supplementary file 1 — Interview guides. This document includes guides that were used to semi structure the in-depth interviews with doctors and patients. (DOCX 21 kb) [file 12913_2019_3876_MOESM1_ESM.docx]

**GUIDE FOR INTERVIEWING DOCTOR at Health Facility**

Objective of interview: To understand provider perspective on organization of health care services for diabetes and hypertension

Thank you for your time, to help me understand how you think and organize health care services for persons with diabetes or hypertension.

Let me begin by asking you a few questions about this health facility you work in

Q. Can you please tell me a little about this place and your work here.

(Probes – private facility/ public, who owns it, what are the terms of their engagement, other doctors, staff, what timings)
Q. How long have you been working here and what motivated you to work here?

Q. What are the major complaints for which people come to you based on your practice and experience here.

You mention diabetes and hypertension –

Q. Is care for diabetes or hypertension part of the general practice or are there special services/ specific days organized for these

Q. Please describe the services available

Probe – for example a person with diabetes or hypertension comes to this facility – then what all would he need to do

Please breakdown” the steps you see in the continuum of care for these chronic patients (e.g. identification of ‘suspects’, diagnosis, start of treatment, follow-up,…)

I would like to know a little more detail on each of the steps you mention (regarding Diabetes and hypertension services)

1. Registration – (probes – who usually does this, is there a card/ book issued, registry, clinical information – how stored and retrieved, how long does this take

what are some of your challenges in organizing this?

2. Laboratory testing (probes – what is available here – glucometer, strips, glycated hemoglobin, when, who does this, how does the report reach the doctor, how is it recorded….

what are some of your challenges regarding lab services?

3. Consultation (Probes – are there other doctors also, how much time on an average would you spend with the patient, what does this include, how do you decide on the medication, how is this recorded, what are some of your considerations, does the patient play a role in decisions regarding treatment

what are some of your challenges in your consultations?

What is your opinion about the use of algorithms or guidelines in day to day practice and decisions to treat and manage?

What do you think of patients and their families being involved in decision making about their treatment.

4. Counseling (probes – who does this, when does this happen, is there any education material provided and what is the purpose of this material, do patients share their concerns, is this recorded, are family members involved

5. Medication (probes – are drugs available here, what are the most commonly used drugs, are these easy to stock, who indents for them, how do you decide which ones to stock, do these get over frequently, are there other pharmacies from where these are accessible –

and what are some of your challenges

6. Payments – how is billing done, is it all together or for each service separately, what would be the average cost for the patient of a routine follow up visit?

Q. You mentioned who performs the tasks involved in each of the steps, who in your opinion plays a substantial role and what sort of role?

Probes – apart from doctor, Is there a team involved in the care?

Probe - What is your opinion of the possibility/feasibility of delegating some (technical) tasks to less qualified health workers and even to patients/care-taker.

Q. How do you screen for complications? Or how do you make sure there are no complications?

(Probe –is it possible in primary care, where, how –appointments, do they return)

Q. How do you deal with complications? What about when referral is required for a complication? To whom would you refer? What are some of your reflections on the process and challenges?

(probe- where, how, do patients return, feedback from referred center, any coordination)

Q. Do you think it is feasible to care for DM/HTN at Primary care?

Probes – diagnosis, starting medication

Q. How do you feel (confidence) in treating diabetes/ hypertension?

Q. How about follow up in general

(how often, what % would you say are regular in the follow up, what would be, you think, possible reasons for patients not to come regularly,

Q. What kind of community resource is available for patients?

Probes - How about the government program, is there any program? If yes, is it useful to you? In what way?

Q. In caring for these patients what are some of your main challenges

Q. What do you think are some things that can be done to make services better

Any other suggestions you may have for the growing burden –in general

7. What is your opinion regarding patient’s self-managing their disease condition

Probes – do you encourage them/ teach them. How often do you call them back, what is your opinion on their ability to do so. What challenges do you face?

Q Do you feel patients are supported in making lifestyle changes?

What are some of your recommendations to improve care at this facility?

**GUIDE FOR INTERVIEWING Patients**

Objective of interview: To understand patient perspectives on organization of health care services for diabetes and hypertension

1. To understand patient’s perspective of their disease and the impact on their lives
2. To understand their challenges and difficulties in accessing care
3. To identify patient expectations from the health facility or services
4. To identify recommendations, they may have to improve the delivery of services

Thank you for giving us time to speak with you today. Our conversation today is for us to understand how it is for you to have diabetes or hypertension, specifically your interaction with the health system and regarding the management of your disease. We will use the information and insights you share with us to improve service delivery for these health conditions and it is for the larger benefit of society.

So, thank you again for your time…please let us know what is a convenient place to sit and talk (here or in your home?)

**Topic one: Personal story**

Q Please tell us how you came to know you had diabetes/ hypertension

Probes – what did you feel, how long ago, who told where to go, where diagnosed, how did you feel, reaction of family, did it affect daily living, how does spouse feel abou this, how do children feel

Q Please tell us a little bit about yourself

Where do you live, what education, what do you do, what does your spouse do, who all in family, how old are children

Q please tell us about your journey since that day of diagnosis

Probes – where all sought care- chronology

**Topic 2 Interaction with the health system for treatment and management**

**Challenges and difficulties**

We would like to know more about the management of this condition

Q Can you describe what happens (process) when you visit the health facility for diabetes/ hypertension

Probes

- lab?, token?
- Waiting time?
- Doctor’s consultation?

Q How do you feel about the interaction with the doctor?

Probes

- How long does he spend – what do you feel about this –(this is enough)
- How do you feel about asking questions/ doubts from the provider…do you feel free or hesitate..
- Can you contact doctor anytime or is it restricted to the clinic visit

Q What all does the doctor tell you…

Probes

- What recommendations/ advice were given in addition to drugs- Any lifestyle changes…….
- Probes - About eating food, salt, veg and fruit, exercise?
- What other sources of information do you have about the disease

Q How do you feel about the recommendations….what did you do about it?

Probes

- Are these easy to follow in your daily routine…why or why not?
- Does your family support you
- Your community- ANMs and ASHAs?
- What is your opinion – should they

Q What is your opinion about why people get diabetes/ hypertension?

Q In your opinion how can it be prevented

Probe- How do you know this

Q How about medication – what has been your experience (availability, affordability)

Probe- Where do you get from?

Q How does the doctor make any adjustments to the treatment – dose?

- Do you feel involved in the decision to change treatment - does he give options available or take your opinion – what is your opinion about this – should they?
- Do you have a book/ card – what is your opinion regarding this

Q Who else do you interact with at the health facility – lab, pharmacy, nurse

- Do they advise
- What is your opinion regarding this – should they

Q How long have you been visiting here

- how often do you have to come
- When was your last visit here?
- How did you decide when to visit again
- any challenges in regular (monthly) visit
- Why do you continue here?
- Why don’t you go to private/ government facility

Q What is good quality care or a good facility

- Some important things in your opinion that make you decide to come here

Q Did you ever have to be referred to another facility for investigation or management of an acute condition?

I would like to know more about this……why were you referred, please tell us in steps

- Was there a slip/ card/ letter
- what you did and where you went…
- how did you feel,
- what advise were you given when you left ?

**Topic 3 Expectations from a health care provider and facility and also community**

Q What are your challenges/ difficulties in living with this disease….

Probe regarding treatment, lifestyle, community interactions

Q How can services be improved at this facility? At the govt/ private

What are your suggestions to help you and other with diabetes or hypertension get along better?
