# Supplementary material for: Challenges in primary care for diabetes and hypertension: an observational study of the Kolar district in rural India
Source: BMC Health Serv Res. 2019 Jan 18;19:44. doi: 10.1186/s12913-019-3876-9 (PMC6339380; doi:10.1186/s12913-019-3876-9)
Supplement: Supplementary file 2 — Observation guide. This was a guide used to provide structure to the non-participant observations. (DOCX 15 kb) [file 12913_2019_3876_MOESM2_ESM.docx]

**NON PARTICIPANT OBSERVATION GUIDE – (outsider at the setting and only observe)**

**Observations to be conducted at health facility**

Objective of observations: To understand organization of health care services for diabetes and hypertension

Detailed note taking for the following

During OPD hours – 10AM to 2PM

At each of the following places in a health facility for minimum of 30 mins each on at least 3 different days or till saturation

1. Registration and waiting area
2. Consultation room (optional depending on facility, doctor and patients) if I am able to do so I would consider spending the entire OPD time in consulting room.
3. Counseling (if applicable)
4. Laboratory
5. Pharmacy

These include individuals’ general appearance (**health facility staff and patients**), verbal and physical behaviors, personal space, human traffic at the observation site, and people who stand out

Observation would end when **theoretical saturation is reached**, which occurs when further observations begin to add little or nothing to understanding. (Liu & Maitlis 2010)

Focus is broad to understand activities at the settings including behaviors but may be narrowed to elements of interest such as interaction of doctor and patient.

Purpose of observation will be known to health facility staff but no explanation to the patients attending the facility. Further researcher’s status as a doctor will only be disclosed to the doctor at the health facility but not to all staff. To the staff status as researcher will be disclosed.

Observation Guide

1. General appearances including age, gender, physical appearance, dress

2. Verbal behavior and interactions among patients and patients with health facility staff: Who speaks to whom and for how long; who initiates interaction; languages or dialects spoken; tone of voice, Roles played by health facility staff

also summarizing content of conversations between staff and patients

3. Physical behavior and gestures: What people do, who does what, who interacts with whom, who is not interacting

4. Human traffic: People who enter, leave, and spend time at the observation site, activities involved in at observation site

5. People who stand out: Identification of people who receive a lot of attention from others and those not receiving attention.

Also, plan a time motion study at each of the sites mentioned above, which involves noting time taken for various activities of at least 2 persons.
