# Supplementary material for: Challenges in primary care for diabetes and hypertension: an observational study of the Kolar district in rural India
Source: BMC Health Serv Res. 2019 Jan 18;19:44. doi: 10.1186/s12913-019-3876-9 (PMC6339380; doi:10.1186/s12913-019-3876-9)
Supplement: Supplementary file 3 — Facility assessment tool. This tool was created using documents (WHO Essential package of services and the Indian public health standards) that describe minimum available resources that should be available at primary level care. (DOCX 102 kb) [file 12913_2019_3876_MOESM3_ESM.docx]

**Facility assessments (tool adapted from WHO essential package of services** [1] **and National standards for PHC** [2])

Instruction: Fill in A for Available and NA when Not available.

For human resource please fill in numbers available.

| Item | Private 1 | Private 2 | Private 3 | PHC 1 | PHC 2 | PHC 3 |
| --- | --- | --- | --- | --- | --- | --- |
| Equipment | | | | | | |
| Thermometer |  |  |  |  |  |  |
| Stethoscope |  |  |  |  |  |  |
| Blood pressure measurement device |  |  |  |  |  |  |
| Measuring tape |  |  |  |  |  |  |
| Weighing machine |  |  |  |  |  |  |
| ECG |  |  |  |  |  |  |
| Defibrillator |  |  |  |  |  |  |
| Laboratory | | | | | | |
| Glucometer |  |  |  |  |  |  |
| Blood glucose |  |  |  |  |  |  |
| Urine protein test strips |  |  |  |  |  |  |
| Urine ketone strips |  |  |  |  |  |  |
| Se Creatinine |  |  |  |  |  |  |
| Troponin test strips |  |  |  |  |  |  |
| Urine microalbuminuria strips |  |  |  |  |  |  |
| HbA1C |  |  |  |  |  |  |
| Blood cholesterol |  |  |  |  |  |  |
| Lipid profile |  |  |  |  |  |  |
| Guidelines | | | | | | |
| Evidence based protocols |  |  |  |  |  |  |
| Indian Guidelines |  |  |  |  |  |  |
| Flow charts with referral criteria |  |  |  |  |  |  |
| Information | | | | | | |
| Medical information register |  |  |  |  |  |  |
| Patient clinic register |  |  |  |  |  |  |
| Audit tools |  |  |  |  |  |  |
| Essential Medicines | | | | | | |
| Thiazide diuretic |  |  |  |  |  |  |
| Calcium Channel Blocker (Amlodipine) |  |  |  |  |  |  |
| Beta blocker (Atenolol) |  |  |  |  |  |  |
| Angiotensin inhibitor (Enalpril) |  |  |  |  |  |  |
| Insulin |  |  |  |  |  |  |
| Metformin |  |  |  |  |  |  |
| Glibenclamide |  |  |  |  |  |  |
| Isosorbide dinitrate |  |  |  |  |  |  |
| Glyceryl trinitrate |  |  |  |  |  |  |
| Furoseamide |  |  |  |  |  |  |
| Aspirin |  |  |  |  |  |  |
| Dextrose infusion |  |  |  |  |  |  |
| Sodium Chloride |  |  |  |  |  |  |
| Human resource | | | | | | |
| Doctor |  |  |  |  |  |  |
| Nurse |  |  |  |  |  |  |
| Lab technician |  |  |  |  |  |  |
| Counsellor |  |  |  |  |  |  |
| Others |  |  |  |  |  |  |

1. WHO. Package of essential noncommunicable disease interventions for primary health care in low-resource settings. 2010.

2. Ministry of Health & Family Welfare G of I. Guidelines for Sub-District/Sub-Divisional Hospitals Directorate General of Health Services Ministry of Health Family & Welfare Government of India. 2012. http://nhm.gov.in/images/pdf/guidelines/iphs/iphs-revised-guidlines-2012/sub-district-sub-divisional-hospital.pdf.
