# Supplementary material for: Challenges in primary care for diabetes and hypertension: an observational study of the Kolar district in rural India
Source: BMC Health Serv Res. 2019 Jan 18;19:44. doi: 10.1186/s12913-019-3876-9 (PMC6339380; doi:10.1186/s12913-019-3876-9)
Supplement: Supplementary file 4 — Coding tree. This is the coding that was used in analysing the data. (DOCX 107 kb) [file 12913_2019_3876_MOESM4_ESM.docx]

| Parent Node | Child Node 1 | Child node 2 |
| --- | --- | --- |
| Profile of doctors and patients | Patient details  Description of doctor | Age, sex,  Age, sex, type of facility,  years of experience, number of years, distance from the town, urban/ rural |
| Care process | Care path  Team and roles  Communication | registration, laboratory, pharmacy, counselling  time taken, time consultation,  cost, screening complications |
| Decision making for care | Guidelines  drugs used  feel about guidelines  Patient involvement | availability  use of guideline  management  affordability |
| Information systems | Continuity  referral,  revisit  reminders | use of book  records at facility |
| Self- management and Coping with condition | Change in lifestyle,  information regarding condition,  when to make a visit decision  compliance  physical coping | Family support,  information doctor give, perceived role in care,  why they did not go back  challenges  routine  Normalization |
| Community support | Health worker, patient groups, neighbors |  |
| Availability of Resources | medicines  manpower  laboratory | Quality of medicines  Affordability |
| Expectation from health care | good care  choice of doctor  perception of government | Communication |

CODING TREE
