# Supplementary material for: Challenges in primary care for diabetes and hypertension: an observational study of the Kolar district in rural India
Source: BMC Health Serv Res. 2019 Jan 18;19:44. doi: 10.1186/s12913-019-3876-9 (PMC6339380; doi:10.1186/s12913-019-3876-9)
Supplement: Supplementary file 5 — Facility assessment. This provides the details of the assessment done for each health facility in the study. (DOCX 120 kb) [file 12913_2019_3876_MOESM5_ESM.docx]

**Facility assessments (tool adapted from WHO essential package of services and National standards for PHC)**

| Item | Private 1 | Private 2 | Private 3 | PHC 1 | PHC 2 | PHC 3 |
| --- | --- | --- | --- | --- | --- | --- |
| Equipment | | | | | | |
| Thermometer | A | A | A | A | A | A |
| Stethoscope | A | A | A | A | A | A |
| Blood pressure measurement device | A | A | A | A | A | A |
| Measuring tape | A | A | NA | NA | A | NA |
| Weighing machine | A | A | A | A | A | A |
| ECG | NA | A | A | NA | NA | NA |
| Defibrillator | NA | NA | NA | NA | NA | NA |
| Laboratory | | | | | | |
| Glucometer | A | A | A | A | A | A |
| Blood glucose | A | A | A | A | A | A |
| Urine protein test strips | A | A | A | NA | A | A |
| Urine ketone strips | A | NA | A | NA | NA | NA |
| Se Creatinine | A | A | A | NA | NA | NA |
| Troponin test strips | NA | NA | NA | NA | NA | NA |
| Urine microalbuminuria strips | A | A | A | NA | NA | NA |
| HbA1C | A | A | A | NA | NA | NA |
| Blood cholesterol | A | A | A | NA | NA | NA |
| Lipid profile | A | A | A | NA | NA | NA |
| Guidelines | | | | | | |
| Evidence based protocols | NA | NA | A | NA | NA | NA |
| Indian Guidelines | NA | NA | NA | NA | NA | NA |
| Flow charts with referral criteria | NA | NA | NA | NA | NA | NA |
| Information | | | | | | |
| Medical information register | NA | NA | A | NA | NA | NA |
| Patient clinic register | NA | Patient retained | A | NA | NA | NA |
| Audit tools | NA | NA | A | NA | NA | NA |
| Essential Medicines | | | | | | |
| Thiazide diuretic | A | NA | A | NA | NA | NA |
| Calcium Channel Blocker (Amlodipine) | A | A | A | A | A | A |
| Beta blocker (Atenolol) | A | A | A | A | A | A |
| Angiotensin inhibitor (Enalpril) | A | A | A | A | A | NA |
| Insulin | A | A | A | NA | NA | NA |
| Metformin | A | A | A | A | A | NA |
| Glibenclamide | A | A | A | A | NA | A |
| Isosorbide dinitrate | A | A | A | NA | NA | A |
| Glyceryl trinitrate | A | NA | A | NA | NA | NA |
| Furoseamide | A | A | A | NA | NA | NA |
| Aspirin | A | A | A | NA | A | NA |
| Dextrose infusion | A | A | A | A | A | A |
| Sodium Chloride | A | A | NA | A | A | A |
| Human resource | | | | | | |
| Doctor | 4 | 1 | 1 | 1 | 2 | 1 |
| Nurse | 10 | NA | NA | 3 | 3 | 3 |
| Lab technician | 3 | 1 | 3 | NA | 1 | NA |
| Counsellor | NA | NA | NA | NA | NA | NA |
| Others | Receptionist also dispenses drugs, ECG technician and nurse |  | ECG Technician, data entry |  |  |  |

A – Available, NA – Not available,
